# Life Depends upon Two Kinds of Water

**DOI:** 10.1371/journal.pone.0001406

**Published:** 2008-01-09

**Authors:** Philippa Wiggins

**Affiliations:** Mairangi Bay, Auckland, New Zealand; Massachusetts Institute of Technology, United States of America

## Abstract

**Background:**

Many well-documented biochemical processes lack a molecular mechanism. Examples are: how ATP hydrolysis and an enzyme contrive to perform work, such as active transport; how peptides are formed from amino acids and DNA from nucleotides; how proteases cleave peptide bonds, how bone mineralises; how enzymes distinguish between sodium and potassium; how chirality of biopolymers was established prebiotically.

**Methodology/Principal Findings:**

It is shown that involvement of water in all these processes is mandatory, but the water must be of the simplified configuration in which there are only two strengths of water-water hydrogen bonds, and in which these two types of water coexist as microdomains throughout the liquid temperature range. Since they have different strengths of hydrogen bonds, the microdomains differ in all their physical and chemical properties. Solutes partition asymmetrically, generating osmotic pressure gradients which must be compensated for or abolished. Displacement of the equilibrium between high and low density waters incurs a thermodynamic cost which limits solubility, depresses ionisation of water, drives protein folding and prevents high density water from boiling at its intrinsic boiling point which appears to be below 0°C. Active processes in biochemistry take place in sequential partial reactions, most of which release small amounts of free energy as heat. This ensures that the system is never far from equilibrium so that efficiency is extremely high. Energy transduction is neither possible and nor necessary. Chirality was probably established in prebiotic clays which must have carried stable populations of high density and low density water domains. Bioactive enantiomorphs partition into low density water in which they polymerise spontaneously.

**Conclusions/Significance:**

The simplified model of water has great explanatory power.

## Introduction

As every biochemistry student knows, enzymes can do anything. With great specificity they catalyse all metabolic reactions; with the aid of small solutes such as ATP and cations, they reverse many spontaneous hydrolysis reactions and accelerate other reactions which, although spontaneous, do not take place *in vivo* in the absence of enzyme. The ‘how’ of these manifold reactions is less obvious. Although the shorthand version of the hypothetical student of biochemistry invoked only enzymes, the operational entities are, in fact enzymes-in-water or, better still, enzymes-in-solution. The chemistry of the surfaces of proteins is well-known. They carry hydrophobic groups, weakly hydrogen-bonding groups such as -C-OH, >NH, -NH2 and charged groups which are the ionised forms of weak acids (-COO−) or weak bases (-NH3^+^), and these charged groups are neutralised by counter-ions. The specificity of the attachment of substrates to such surfaces arises from geometrical matching of hydrogen bond donors and acceptors, of opposite dipoles and of opposite charges. It is hard, however, to imagine how the conformational changes that the proteins undergo during activity can transform these weakly-interacting groups into the aggressively reactive state required for some enzymic processes. Just four reactions have been chosen to illustrate this problem.

### Energy transduction

Energy transduction says that the free energy of hydrolysis of ATP or of dissipation of a cation gradient can be harnessed by enzymes to do work. In fact the free energy of any spontaneous reaction is always dissipated as heat. Reactions are coupled, in a thermodynamic sense, only when the product of one reaction is a reactant of the second. So the energy required to transport cations against gradients or to make the filaments slide or to synthesis ATP or peptides or polynucleotides or to perform any of the reactions idiosyncratic of life, is not a transduction of the free energy of hydrolysis of ATP. That hydrolysis of ATP is an essential part of the overall reactions of cation pumps and motor molecules is not in question, but the mechanism is. Since water is a reactant in these reactions, and is also the solvating medium in which they take place, its involvement is inevitable. Phosphorylation of the enzyme by ATP must change the local environment in which the reaction takes place, so that its free energy change becomes negative. That environment is water close to the enzyme surface.

### Hydrolysis of peptides and polynucleotides

Proteases and Dnases hydrolyse peptide bonds and oligonucleotide bonds, respectively, at neutral pH and 38°C or ambient temperature. To break these bonds *in vitro* requires boiling in 6M HCl. Somehow these enzymes-in-solution have acquired the properties of hot concentrated strong acids. Again it is impossible to ignore a contribution of water, which is present and is required for the development of acid properties.

### Formation of bone

The mineral component of bone is Ca_3_(PO_4_)_2_. *In vivo* this forms at pH 7.4 and 38°C from the normal concentrations of calcium and phosphate (approximately 2 mM) in blood. Similar ceramics require a temperature of 1200°C, presumably, to dry the product. The concentration of the species PO_4_^3−^ in the blood is probably of the order of 10^−6^ M, making it far too dilute to precipitate out at all. Indeed, we know that the solubility product is not exceeded in blood because bone forms only in conjunction with specialised cells (the osteoblasts). Crystallisation of Ca_3_(PO_4_)_2_ is a process which depends entirely upon the solvent, which is water, and the electrostatic attraction between Ca^2+^ and PO_4_^3−^. Only changes in the solvent properties of water could allow crystallisation of bone at pH 7.4 and 38°C.

### Sodium and potassium

Many enzymes discriminate with exquisite precision between sodium and potassium, a feat which appears beyond the most green-fingered chemist. Carboxyl groups show a slight preference for K^+^ over Na^+^ (2∶1) but nothing approaching the 30∶1 of the Na,K-ATPase and the many other enzymes which absolutely require either K^+^ (eg pyruvate kinase) or Na^+^ (eg Na^+^-dependent active transporters). A scrutiny of the aqueous solution chemistry of the two cations shows that their only significant difference lies in their effects upon the water in which they are dissolved. Na^+^ slightly increases water structure and K^+^ slightly decreases water structure.

### Can water change its physical and chemical properties?

Table 1 shows the boiling points of the hydrides of oxygen and its neighours in the 1^st^ row and 6^th^ group of the Periodic Table. Neighbouring hydrides in the first row are all gases at ambient temperatures; they boil well below 0°C, the freezing point of water. As one moves up the hydrides of Group 6a elements from Te to S, their boiling points progressively decrease. Extrapolating these boiling points to the molecular weight of water, gives an expected boiling point of about −75°C. These figures were first produced by Henderson [1].

**Table 1:** Boiling points of Hydrides near Oxygen in the Periodic table

Group	3A	4A	5A	6A	7A
	B_2_H_6_; −92.5	CH_4_; −164	NH_3_; −32.4	H_2_O; **+100**	HF; −87.7
				H_2_S; −60.7	
				H_2_Se; −41.5	
				H_2_Te; −2	

There is obviously something very strange about the boiling point of water. We now know that the strangeness is a consequence of the extensive three-dimensional hydrogen-bonding of water molecules to each other; breaking the residual hydrogen bonds in the liquid requires an abnormally high temperature. Reverting to the hydrides of the first row, however, we note that NH_3_ and HF, which also mutually hydrogen bond, still have much lower boiling points than does water. The explanation is that the strength of water-water hydrogen bonds greatly exceeds that of intermolecular bonds in NH_3_ or HF. The question that heads this section, therefore, can be answered with a qualified yes. If water, somehow, locally changed its density so that its hydrogen bonds became straighter and stronger or bent and weaker, all its physical and chemical properties must change. Its boiling point would decrease as bonds became weaker, free energies of hydration of Na^+^ and K^+^ and of other cations and of reactants and products of hydrolysis reactions must all change. An increase in hydrogen-bond strength must lead to the opposite changes in physical and chemical properties.

### Does water change its properties at surfaces?

In order for these various processes to take place, the changes in the structure of water and, therefore, of its properties must extend over a zone large enough to accommodate the reaction species. Before the early 1990s no plausible mechanism for the formation of appreciable volumes of modified water had been suggested. Ling [2] had recognised this problem and proposed that intracellular water existed as polarized multilayers at protein surfaces, thus providing adequate volumes of modified water. It has generally been considered, however, that hydration of a protein surface is not long-range. At a protein surface, some water molecules must interact directly to hydrate the surface, but there is no reason to expect that this would involve more that one or at the most two, layers of water molecules. The dominant water-water bonding must then take over, if water consisted, as was generally believed, as a single continuous three-dimensional hydrogen-bonded network in which water-water bonding was, in general, stronger than water-surface bonding [3]. In spite of this theoretical void, the clear need for mechanisms in the neglected areas of energy transduction, hydrolysis of peptides, mineralisation of bone and discrimination between Na^+^ and K^+^, encouraged a search for modified water inside cells and at surfaces. Both high density and low density water were found, but not consistently. In many experiments no change was detected in the properties of water at surfaces; when changes were detected they could only be interpreted in terms of an accepted model of water structure. That model was, usually, normal bulk-phase water together with water “bound” to surface moieties. This limited the usefulness of the experiments which had found modified water. The field was obviously ripe for an imaginative leap of understanding. But, of course, feasible leaps of understanding cannot be produced to order. The trick is to recognise one wherever and whenever it arises.

When it came, in the early 1990s, this imaginative leap came from physics [4]–[10], not biology. It turned out to have the attributes of a good testable hunch. It was simple and limited in scope (proposing the coexistence of just two types of water in rapidly exchanging microdomains). It offered many testable predictions, some of which satisfied the requirements for enzyme reactions already perceived; others were a bonus. For example, it was found that a solute which partitioned preferentially into low density water (LDW) (eg. K^+^) created local osmotic pressure gradients which could only be abolished by conversion of LDW to high density water (HDW). This immediately suggested a well-documented mechanism of enzymes. Enzymes start in an inactive state, are converted to an active state which performs the characteristic reaction, but must then revert to the inactive state in order to be able to repeat the cycle. If the active state contained LDW, then K^+^ would demolish it and restore the inactive state. This mechanism by which small solutes demolish their own preferred environment has proved of general application.

### The mixture model

Three independent lines of inquiry have converged on the proposal that liquid water can exist as two different polymorphic structures. Such interdisciplinary convergence adds greatly to the plausibility of the proposal.

### The model of GW Robinson and coworkers

Robinson and coworkers showed that all the anomalies of liquid water could be accounted for quantitatively in terms of microdomains of different density [4]–[6] Many of the anomalies, first listed by Henderson in 1913, have since been shown to be a consequence merely of the cohesive character of water; i.e. that water exists as networks of molecules interconnected by hydrogen bonds. For example the remarkably high melting point and boiling point of water can be explained in this way. When it is compared with other hydrides of Group 6 of the Periodic Table (H_2_S, H_2_Se, H_2_Te) which are not mutually hydrogen-bonded, the surprising prediction is that non-bonded water should melt at about −95°C and boil at about −75°C. In addition to these phenomena which are compatible with any cohesiveness, there is a group of anomalies which is more demanding. These have yielded to Robinson's two-state water. They include a maximum in density at 4°C, a minimum in compressibility at 46.5°C and a decrease in viscosity with increasing pressure below 33°C. Moreover D_2_O and T_2_O differ from H_2_O more than predicted from cohesiveness alone.

**Figure 1:**
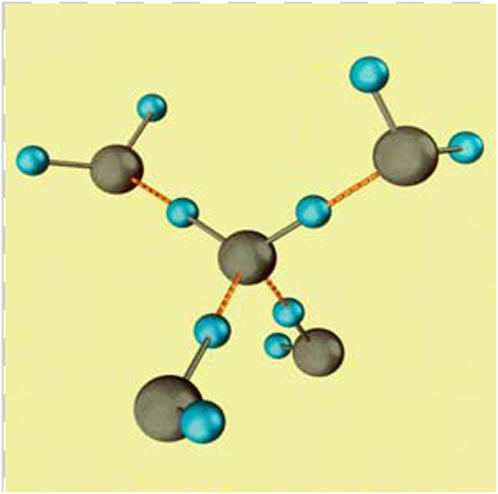
A cluster of five water molecules as they would appear in ice1h or transiently in LDW. Each oxygen is linked to two hydrogen atoms to which it is covalently bonded and to two more, on other water molecules, to which the central oxygen is hydrogen-bonded. In LDW each H atom lies on a straight line between two O atoms.

Figure 1 shows the structure of ice 1h and of LDW. In LDW each H atom lies on a straight line between two O atoms. HDW is a collapsed form in which the hydrogen bonds, which in LDW keep molecules apart, are bent and allow molecules to approach each other and increase density. These bent bonds are relatively weak.

### The model of HE Stanley and coworkers [7]–[10]

The most interesting region of liquid water lies in an experimentally inaccessible temperature range below −40°C, the homogeneous nucleation temperature. Experimentally, it is impossible to prevent pure water from crystallising to ice. Computer simulations, however, can impose constraints that prevent freezing, so that the liquid survives to lower temperatures. At about −50°C, liquid water surpasses itself in strange behaviour and separates into two liquid phases of different density, a high density form and a low density form. Of course, this is computer water. It does not necessarily follow that real water would, if it could, do the same thing. But Stanley and coworkers have shown that as cooling continues, high and low density liquid waters pass smoothly into high and low density amorphous ices. These ices have been well investigated, both experimentally and in their electronic state. They really exist. Again, the estimates of density are about 0.91g/ml and 1.2 g/ml, a remarkable difference of 30%. The corollary of this separation into two liquid phases is that, with computer water, at least, warming through −50°C should reveal merging of the two liquids into one, so that, like Robinson's model, microdomains would coexist to higher temperatures throughout the liquid range. Chaplin [11] has suggested that water has expanded and collapsed structures based on a 5-fold symmetry which limits growth of microdomains to about 3 nm diameter.

### Evidence from biology

The third line of evidence comes from a scrutiny of biological processes, especially enzyme reactions. Like the outstanding anomalies of the pure liquid, a full mechanistic explanation seemed to require more than the random hydrogen bonding of one-state water. In particular, the ability of some enzymes to hydrolyse ATP, peptides and polynucleotides and others to synthesise ATP and biopolymers, a pervading phenomenon in biochemistry, had no detailed molecular mechanism. The concept of energy transduction which was proposed in the 1960s has survived to achieve the status of a textbook fact, but it does not constitute a molecular mechanism. The free energy change of a reaction indicates how far the reaction, as written, is from equilibrium and, therefore, whether or not the reaction will proceed spontaneously. For example, the free energy change of the reaction:
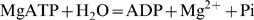
is approximately −30 kJ/mol. Here Pi means the mixture of H_2_PO_4_^−^/HPO_4_^2−^ that exists at the prevailing pH. This is a rather high negative free energy change which means that the reaction, as written, will go spontaneously from left to right, and that the reverse reaction from right to left has a free energy change of +30 kJ/mol and will not go spontaneously. The magnitude of the free energy change shows that the reaction is far from equilibrium. As the reaction proceeds from left to right, 30 kJ/mol of energy is dissipated as heat. The concept of energy transduction states that the negative free energy of a spontaneous reaction, such as hydrolysis of ATP, can be used to drive an uphill reaction, such as movement of cations from low to high concentrations, provided that the two reactions are coupled in a single enzyme active site [12]. The authors take the case where the uphill reaction has a positive standard free energy change:
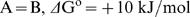
The spontaneous reaction has a larger negative standard free energy change
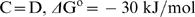
When the two reactions are coupled the overall reactions is:
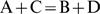
and the free energy change is ΔG^o^ = +10–30 = −20 kJ/mol i.e. when the reactions are coupled, the sum of the two standard free energy changes is negative, and the uphill reaction can proceed. The uphill reaction might be movement of Na^+^ from a low concentration inside a cell to a higher concentration outside the cell, and the spontaneous reaction hydrolysis of ATP. This is called energy transduction. It is taken to mean that free energy stored in the bonds of ATP can be converted to other forms of energy and harnessed to do work. This concept was vigorously debated in the 1960s. Physical chemists pointed out that, however and wherever ATP was hydrolysed, the free energy of that hydrolysis was dissipated as heat and could not be diverted to or bestowed upon another unrelated reaction, however hungry that reaction might be for kJ [13]. True coupling of reactions requires that a reactant of the uphill reaction is a product of the spontaneous reaction, so that it is so rapidly scavenged that the reaction can proceed. Biochemists, in their turn, pointed out that whenever the two reactions occurred together in the active site of the sodium pump, ATP was hydrolysed and work of transport performed. Without hydrolysis of ATP no transport occurred. The fallacy in their argument was that they believed that each step in the overall process must have a negative free energy change. In fact, the criterion that a reaction will proceed is simply that the free energy of the products is more negative that that of the reactants. There can be ups and downs on the way from reactant to products, but as long as all the steps considered contribute to the overall unitary process, all that matters is the beginning and the end, irrespective of how the system gets there.

### Resolution of the conflict

The solution to this controversy was clearly that both were partially right: that ATP was hydrolysed and that free energy dissipated as heat, and that ATP hydrolysis was necessary for the work of transport to occur. Dissipation of the free energy of hydrolysis as heat does not detract from its essential role in allowing the unitary reaction of ATP hydrolysis and work performance to proceed. All that is necessary is that the free energy of the products is more negative than the free energy of the reactants. This has been shown to be true by many calculations. The first step in these transport reactions is phosphorylation of the enzyme by ATP [14]–[16]. It must follow that, by phosphorylating the enzyme, ATP induces a change in the local environment of the active site which allows the uphill reaction to proceed. This was a particularly good example to take, because, in the reverse running of the pump the same phosphoenzyme synthesised ATP from ADP, suggesting that the same changed environment allowed both the uphill Na^+^ transport and the uphill synthesis of ATP to occur. The debate, however, fizzled out, with biochemists emerging as winners by default. A key paper by one of the groups participating in the debate gave a very good clue as to the kind of local environmental change that phosphorylation of the enzyme caused. George *et al* [13] showed that the free energy of hydrolysis of molecules like ATP was given by the difference between the free energies of hydration of products and reactants. They called this paper “Squiggle-H_2_O. An enquiry into the importance of solvation effects in phosphate ester and anhydride reactions”. At this time the terminal phosphate bond of ATP was called a high-energy bond and given the symbol ∼P. The “Squiggle-H_2_O” paper showed that there was nothing special about squiggle P but that the hydration free energies of all components of the reaction were of crucial importance. This immediately suggested a way out of the impasse reached between physical chemists and biochemists. Phosphorylation of the active site of the pump protein somehow changed the hydrogen-bonded structure of water inside that cavity. Changes in water-water bonding must change all free energies of hydration of solutes in that cavity. Some ions might move out of water which was less able to hydrate them (eg. Na^+^, H^+^, Ca^2+^) and both the sign and the magnitude of the free energy of hydrolysis of ATP might also change so that synthesis became a spontaneous process. These changes in hydration free energies are necessary consequences of a change in local water-water bonding, because the first step in the solution of a solute is making a hole in the water big enough to accommodate it. This involves breaking a considerable number of water/water bonds. The free energy required to break bonds must change with the water structure.

Subsequently experiments showed that volume was, indeed, important.

For example cations show a preference for LDW in the order: Cs^+^>K^+^>Na^+^>Li^+^>H^+^. The break occurs between K^+^ and Na^+^; i.e. K^+^ is a chaotrope and Na^+^ is a kosmotrope. Univalent anions show the same trends. Their order is I^−^>Br^−^>Cl^−^>F^−^. Here, the break is between Cl^−^ and F^−^. The only differences between the ions on either side of the break is size.

At this stage the proposal that water in the enzyme active site assumed stronger water-water bonds when the enzyme was phosphorylated was highly speculative, and, of course, unpopular [17]. Unlike energy transduction, however, it was thermodynamically possible. Even at that time there were reasons to suppose that water at surfaces was perturbed relative to bulk water. Moreover, as more experimental data accumulated, the explanatory power of the hypothesis made it increasingly plausible. It fitted very nicely with the rule of thumb laboratory practice of organic chemists that a poor yield of a reaction could often be improved with a different solvent.

### ATP the energy currency of the cell

ATP hydrolysis is associated with a very large number of reactions which cannot proceed spontaneously but do so when accompanied by ATP hydrolysis I [18]–[23] These include uphill transport of Na^+^, H^+^, and Ca^2+^, driving motor molecules, folding proteins, and synthesising peptides, polynucleotides and polysaccharides. It became clear that the change in microenvironment of these enzymes upon phosphorylation or binding of ATP must be of a very general nature. Ions transported uphill (Na^+^, H^+^, Ca^2+^) were all highly hydrated and of the class of ions which had long been known to increase water structure, whereas K^+^, which is pumped into rather than out of cells was a water-structure breaker. Other examples of “energy transduction” were synthesis of ATP (an uphill reaction) enabled by flux of cations downhill (a spontaneous reaction) [19]. In the ATP synthase the flux was of H^+^; in the reverse running of the Ca^2+^ pump the flux was of Ca^2+^ ions and in the reverse running of the Na^+^ pump, the flux was of Na^+^. Of course, the free energy changes of these spontaneous fluxes of cations were dissipated as heat. Again there must be a molecular mechanism whereby ATP is synthesised in the changed environment of the enzyme active site, and, perhaps released by a flux of cations.

The status of the concept of energy transduction in biochemistry is probably one consequence of the specialisation of scientists in the last half of the twentieth century. Langmuir, when asked, called himself a scientist, not a physicist or physical chemist. Pauling, though known as a chemist first and foremost, made extensive contributions to biology. In the 1960's, physical chemists, such as George [13], were familiar with the growing biological literature and could contribute with authority to the thermodynamics of active transport. Now, most physical chemists are not aware of the thermodynamic problems hidden in biochemistry; and biochemists learn their thermodynamics from biochemists.

### The experimental strategy

The amount of water contained in the active sites of enzymes is so small that its direct characterisation was very difficult. Any water-related property must be swamped by the simultaneous presence of excess bulk water. Experiments with actomyosin, the Ca-ATPase of sarcoplasmic reticulum and mitochondria are discussed elsewhere [23].

A corollary of the proposal for water with stronger water-water bonds in enzyme active sites was that such water must also exist elsewhere. Vital water, as a scientific explanation, was not thinkable. Our experiments, therefore, concentrated on non-living water-filled microporous systems with cavities 1–3 nm across lined with protein-like surfaces; i.e. with weakly hydrogen-bonding, hydrophobic and charged moieties. Details of the methods are given in the referenced papers.

### Difficulties of interpretation of these experiments

Before giving some results of these experiments, it is fair to say that early results were often most unexpected and difficult to explain in terms of the conventional view of water structure. They, therefore, contributed to a strong interest in the new mixture model of water, and directed subsequent experiments. The main thrust of this new interest, however, was a theoretical exploration of the consequences of coexisting HDW and LDW and especially their role in biology.

### Solutes and two-state water

When a solute is added to single-state water all environments are the same. But in two-state water there are two environments which are profoundly different, as experiments have revealed. Any solute, therefore, partitions more-or-less differently between contiguous microdomains. The consequence is that there are instantaneous gradients of water activity between contiguous unlike microdomains.

In two-state water there will always be potential local gradients in water activity. In solutions where there are no barriers to diffusion they must be avoided, eliminated or compensated for in some way. Local gradients can be eliminated by converting LDW microdomains into HDW microdomains until all concentrations are equal. In this configuration, however, water is in equilibrium but solute is not. The configuration arrived at by the solution of chaotrope is that of lowest overall free energy. It seems that in this configuration neither water nor the solute is in its state of lowest free energy. That configuration will include a reduction in the osmotic pressure gradients by conversion of some LDW into HDW with redistribution of solutes. But if all water was converted to HDW by solution of a chaotrope, all chaotropes would have the same effect upon water structure. This does not happen. There appears to be a rank order of chaotropes and their effect upon the entropy of water. A similar mechanism must apply to solutes which partition preferentially into HDW. In order to eliminate standing osmotic pressure gradients, some HDW microdomains must become LDW microdomains. Again, there will be a single configuration of lowest free energy that will depend upon the magnitude of the partition coefficient, and on temperature and pressure.

This gives rise to a biological equivalent of Heisenberg's Uncertainty Principle. Measurement of partitioning of solutes between HDW and LDW destroys the type of microdomain that they prefer. Therefore there are no neat lists of solutes and partition coefficients to delight the seeker after “hard numbers”. Partition coefficients depend upon all components of a system, including the concentrations of the solutes themselves.

### Cost of displacement of the HDW/LDW equilibrium

An important mechanism that emerges from the concept of induction and elimination of osmotic pressure gradients in solution, is that displacement of the equilibrium in either direction incurs a thermodynamic cost. This follows because the position of equilibrium in pure water is set by the prevailing temperature and pressure. Its displacement introduces a positive term into the overall free energy of hydration. If this term is sufficiently large, the free energy of hydration may become positive and limit the solubility of the solute. This thermodynamic cost evidently prevents HDW from boiling at its intrinsic boiling point which Table 1 suggests is probably well below 100°C. If it boiled at, say, 20°C, residual water would become increasingly enriched in LDW. Under most sets of conditions, therefore, it does not boil until LDW boils at 100°C.

As in most water-related changes, enthalpy/entropy compensation plays a dominant part. This means that any change in the water equilibrium can involve rather large changes in entropy and enthalpy but that, because these tend to cancel each other out, (ΔG = ΔH − TΔS) the resulting change in free energy is relatively small. It depends, of course on pressure and temperature (which determine the position of the equilibrium) and is, in general, greater for conversion of HDW into LDW, because the PΔV term is positive going from HDW to LDW, but negative going from LDW to HDW.

The existence of this thermodynamic constraint determines many properties of the mixture model of water. For example: it prevents evaporation of HDW which, according to Table 1, should have a boiling point much lower than 100°C; it inhibits ionization of water; it drives folding of proteins; it largely determines solubility.

### Partition of neutral salts

The rank order of partitioning of single ions has been deduced qualitatively from the behaviour of neutral salts in cellulose acetate membranes and polyamide beads. Absolute values are not attainable, because the positive and negative ions of a salt in solution cannot be separated and their individual properties obtained. Both ions of a neutral salt must occupy the same type of microdomain determined by the relative potencies of the individual ions as chaotropes or kosmotropes. For example, CaSO_4_ is sparingly soluble partly because both Ca^2+^ and SO_4_^2−^ are potent kosmotropes, requiring a large displacement of the water equilibrium. CaCl_2_, on the other hand is highly soluble, partly because Cl^−^ is a chaotrope, so that the displacement of the water equilibrium by Ca^2+^ is corrected by an opposite displacement by 2Cl^−^. It turns out that both CaCl_2_ and CaSO_4 _partition into HDW but CaSO_4_ much more strongly than CaCl_2_. The other factor of course that determines solubility of a salt is the force of attraction between the ions in the crystal.

### Free energies of hydration of single ions

As pointed out above, free energies of hydration of single ions cannot be measured directly because a single ion cannot be separated from its partner in a conducting solution except by the application of a high electric field. Nevertheless lists of free energies of hydration of single ions have been published. They are deduced from the experimental free energies of hydration of neutral salts, usually with some extra-thermodynamic assumption. For example, it was often assumed that the hydration characteristics of K^+ ^and Cl^−^ were the same and that the free energy of hydration of KCl was equally divided between the two ions [24]. With this assumption together with the further implicit assumption that the free energy of hydration of an ion was a constant quantity, independent of the identity of its companion ion or ions, free energies of hydration of other cations were calculated from the free energies of hydration of their chlorides and free energies of hydration of other anions from their K^+^ salts.

### What determines whether a particular solute prefers LDW or HDW ?

When a solute dissolves in water, there are three components of the free energy of hydration:

1. water-water hydrogen bonds must be broken to make a hole into which the solute fits
2. water immediately adjacent to the solute molecule must reorient itself as it interacts with the solute molecule
3. the HDW/LDW equilibrium must shift so that water in contiguous microdomains is restored toward equilibrium.

Of these three terms, each increases in absolute magnitude as the volume of the solute increases and thus increases the extent of the interface between solution and water. ΔG_1_ is always positive as it involves breaking hydrogen bonds and increasing the entropy of water; it is greater for solution in LDW than for solution in HDW. ΔG_2_ is the negative term which confers solubility upon the solute. It is of greatest magnitude for ionic kosmotropes; and of least magnitude for nonionic kosmotropes. ΔG_3_ is also positive because it involves displacing the water equilibrium at constant temperature and pressure and is of greatest magnitude for solution of kosmotropes.

### Biological solutes in two-state water

Apart from ions, which can have extreme individual preferences for either HDW or LDW, most biological solutes have relatively modest partition coefficients. This is because they have moieties which are chaotropic (-OH, NH2, COOH), and moieties which are kosmotropic (C, CH, CH_2_ in aliphatic and aromatic molecules). Since a molecule can be in only one place at a time, the overall preference for either HDW or LDW and the cost of abolishing the osmotic pressure gradient are both slight. These molecules are characteristically very soluble. Proteins, also, have mixed moieties, and, on the whole are more soluble than synthetic polymers. Folding of proteins is a response to the need to reduce the surface area of contact between protein and water in order to escape a high thermodynamic cost of displacing the water equilibrium.

### Salting in and salting out of proteins

Hofmeister [25] first showed that some neutral salts increased the solubility of proteins while others decreased solubility or were ineffective. The conventional view is that proteins and salts competed for water of hydration. The specificity of effects is more readily explained in terms of two-state water. A protein precipitates out when the thermodynamic cost of hydrating it becomes too high. Proteins are made up of both kosmotroic and chaotropic moieties. If kosmotropic moieties predominate, precipitation of the protein will occur when the free energy of induction of LDW becomes too high. This could be offset by a chaotrope which induces HDW, decreasing the overall cost of displacing the water equilibrium. One would expect, therefore chaotropic solutes to increase the solubility of kosmotropic proteins and kosmotropic solutes to increase the solubility chaotropic proteins. This mechanism is compensatory rather than competitive. Like many phenomena of aqueous solution chemistry salting out of proteins is readily explained by the competition between solutes and proteins for water of hydration. Salting in, however, requires two-state water to explain how the solubility of a protein can be enhanced by addition of specific other small solutes

### Surfaces and two-state water

Surfaces, also, induce osmotic pressure gradients in two-state water, since chaotropic moieties on the surface tend to induce HDW and kosmotropic moieties tend to induce LDW. So far, surfaces are similar to solutes. In addition, however, surfaces induce osmotic pressure gradients in several modes that are not operative with small solutes. This leads to new levels of complexity.

### Biological surfaces and two-state water

Biological surfaces are never smooth on an atomic scale. For example, proteins have side-chains extending from the backbone of the molecule into surrounding water. A zone of water immediately adjacent to the backbone and containing those side chains, has, consequently, a lower activity than a zone of similar size in the bulk solution. Water cannot move in toward the surface to abolish this water activity gradient, because the side-chains are covalently bonded to the backbone. This is an example of a compensatory mechanism in which the gradient of activity of water cannot be abolished by movement of water. As in classical osmotic theory, if there is a residual osmotic pressure gradient there must be a pressure gradient. In this case the pressure gradient is caused by water itself which tends to move in to abolish its activity gradient and is prevented. The pressure gradient, therefore is always directed inward toward the surface, so that there is a positive pressure acting on the water at the surface and a negative pressure acting on more distant water.

The important difference from classical osmotic theory is that in two-state water pressure gradients displace the water equilibrium, either inducing HDW where the pressure is positive and/or inducing LDW where the pressure is negative. Whether the compensation for the osmotic pressure gradient predominantly induces HDW or LDW depends upon the position of the equilibrium before application of the gradient. If water at the surface is already predominantly HDW, then the pressure gradient will induce principally LDW. This is a source of many apparent paradoxes.

### Charged surfaces and two-state water

A surface carrying a fixed charge has an excess concentration of counterions in a zone adjacent to the surface, creating another osmotic pressure gradient, which, again, cannot be abolished by movement of water, but acts as a pressure gradient oriented in the same direction as the gradient due to surface roughness. Again, it is independent of the chemistry of the surface moieties, other than their charge, and of the chemistry of the counter-ion. The fixed charges on biopolymers are always strong chaotropes (large univalent anionic or cationic groups), inducing HDW in the double layer. Experiments with glass beads showed that counterions to fixed charges had powerful and extremely specific effects upon local water structure. Added solutes also have effects other than that of the counter ion. Excess solutes can change the thickness of the double layer by changing the dielectric constant. When the double layer increases in thickness the excess concentration of counterions decreases, as do all the secondary effects of that osmotic pressure gradient. Conversely a decrease in its thickness amplifies all the osmotic effects and their consequences.

### Superposition of the several compensations for osmotic pressure gradients

The five different modes of compensating for standing osmotic pressure gradients sometimes reinforce one another and sometimes oppose one another. The two gradients which are essentially properties of the surface and independent of the chemistry of that surface always tend to induce HDW immediately adjacent to the surface and LDW further out. The gradient due to the specific chaotropic properties of the fixed charges has the same effect. When the counterion is also a chaotrope the cumulative effect is such that water adjacent to the surface appears to become pure HDW and compensation for the pressure gradient consists predominantly in induction of LDW. This, also, leads to many paradoxical observations. For example chaotropes can act as kosmotropes and *vice versa.*

These considerations are of great biochemical import, because all biopolymers are polyelectrolytes which interact with small solutes in these apparently eccentric ways.

## Results

It is not possible to probe a single kind of water in the bulk liquid: any water-related property is an average value. It has turned out, however, as predicted theoretically, that it is possible to sample a single type of water at surfaces. Individual properties, therefore have been obtained from experiments with the following surfaces.

### Cellulose acetate membranes [26]

Salts which, above, have been classed as kosmotropes (NaCl, LiCl, CaCl_2_, MgCl_2_) were, in that order, increasingly excluded from the pores of the membranes. Exclusion increased with increasing concentration. KCl and CsCl were accumulated from very low concentrations (of the order of 0.1 mM) but at concentrations as low as 10 mM there was no significant difference between their concentration in the external solution and that in the pores.

**Figure 2:**
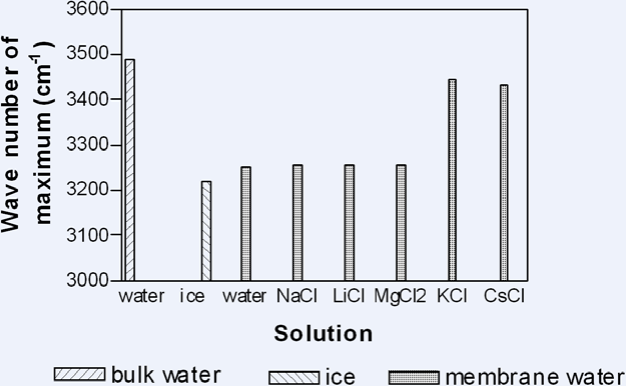
The positions of the maxima of the OH stretch band in water inside dense cellulose acetate membranes equilibrated with water and various solutions.

The OH stretch band of the infrared spectrum of water in dense cellulose acetate membranes soaked in water peaked at 3200 cm^−1^, the value for ice. (see Figure 2). The height of this peak increased in the presence of the excluded salts and increased with the degree of exclusion. This was all consistent with the presence of LDW inside the pores. The behaviour of the pore water in the presence of KCl and CsCl was the first intimation of oscillations which have been identified in many systems. At extremely low concentrations of KCl and CsCl, the OH stretch band still peaked at 3200 cm^−1^ and the ions were accumulated, but at the higher concentrations when the ions were not accumulated, the OH stretch band peaked at 3400 cm^−1^, the value for liquid water. This suggests that the strongly cross-linked matrix of cellulose acetate had very little flexibility and offered great resistance to all but a very slight degree of swelling.

In a rigid cavity or in a flexible cavity which has reached the limit of its capacity to swell, further accumulation of KCl creates an osmotic pressure gradient which cannot be eliminated by movement of water, and is eliminated by switching of LDW to HDW inside the cavity. When this occurs, KCl has destroyed its own preferred environment and diffuses spontaneously out, eliminating the osmotic pressure gradient. LDW reforms inside the cavity and the cycle is repeated. The frequency of these oscillations depends upon the flexibility of the walls of the cavity and also on the presence or absence of solutes which, by virtue of size or solvent selectivity are excluded from the cavity. Oscillations have been followed with time by measurement of the internal water of a gel. In polyamide beads the frequency of oscillations was of the order of days (see under polyamide beads)

Oscillations in cellulose acetate membranes were recognized by the OH stretch band at 3400 cm^−1^: it appears that the water spent some time as LDW (3200cm^−1^) and some time as HDW (probably 3600 cm^−1^) which averaged out at 3400 cm^−1^. It is no coincidence that this is the same as the OH stretch band of pure water, if pure water consists of a mixture of LDW (3200 cm^−1^) and HDW (3600 cm^−1^).

That we were dealing with osmotic pressure gradients was confirmed by experiments which showed that internal KCl concentration could be increased by coequilibration with an excluded solute, such as MgCl_2_, betaine or butanol. It appeared that destruction of LDW by KCl could be prevented if it was balanced by an excluded solute, making the activity of water the same in the two compartments

These results, together with many other results in different systems, illustrated some important principles: If water can move to eliminate an osmotic pressure gradient, it will move. That is always the first choice. Thus in the presence of an extremely low concentration of KCl or CsCl, accumulation of the ions into the internal LDW was accompanied by water, abolishing the osmotic pressure gradient, and allowing retention of the LDW . This requires, of course, that the water that entered the pores to eliminate the osmotic pressure gradient, was, or became LDW.

This is a second important principle which has many examples. A polymeric surface such as cellulose acetate is not smooth: there are projecting hydrophobic elements which, when they can, fold down on to the surface, reducing the area of contact between hydrophobic surface and water and avoiding a costly displacement of the HDW/LDW equilibrium. When, however, more water enters to abolish an osmotic pressure gradient, some elements detach from the surface, and the incoming water becomes LDW. This is possible only if the pore can expand to accommodate the extra water. Therefore pores expanded slightly at low concentrations of KCl, but were unable, because of their cross-linked structure, to expand enough to accommodate the amount of water needed to abolish a large osmotic pressure gradient. So 10 mM KCl continued to accumulate in the pores after the capacity of the pores to expand had been reached. The only choice left was for the water inside the pores to switch to HDW. That immediately stopped accumulation of KCl and initiated a total efflux. When the efflux of KCl was complete, water in the pores reverted to its LDW state. This had two experimental results: the concentration of internal KCl was not very different from its concentration ion the external solution, and the OH stretch band peaked at 3400 cm^−1^ because it oscillated between 3200 cm^−1^ while the water was LDW and 3600cm^−1^ while the water was HDW.

### Synthesis of ATP from ADP and KH_2_PO_4_ in cellulose acetate films

In the presence of 100 mM NaCl or of 100 mM MgCl_2_ no ATP was detected. In the presence of 100 mM KCl or of no additions other than the 5 mM potassium phosphate as reactant, ADP was converted quantitatively to ATP. The sum of ADP plus ATP remained constant while ATP increased and ADP decreased over an interval of several days.

The conclusion was that the synthesis is a spontaneous reaction in LDW, but that the release of chaotropic ATP from the membranes required conversion of LDW to HDW. This was achieved by potassium phosphate or by added KCl, but was opposed by addition of 100 mM NaCl or 100 mM MgCl2, either of which stabilised LDW and prevented release of ATP. This requirement is consistent with the earlier findings that cellulose acetate pores have little flexibility and do not allow influx of much water.

The relationship between work performance and energy consumption is best understood by reducing this simple unitary process of:
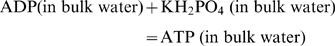
to a series of sequential partial reactions:

1. membrane is added to ADP and KH_2_PO_4_ in water
2. water diffuses into pores and becomes LDW
3. ADP and KH_2_PO_4_ diffuse into LDW in pores,
4. K_2_HPO_4 _remains in the external solution: the two osmotic pressure gradients cancel each other.
5. the reaction ADP + KH_2_PO_4_ = ATP takes place
6. more KH_2_PO_4_ or KCl diffuses into the pores, overcoming the osmotic pressure gradient due to exclusion of K_2_HPO_4_
7. since the pore has reached the limit of its expansion, LDW converts to HDW
8. ATP, ADP and KH_2_PO_4_ diffuse out into external water.

Of these partial reactions, 1, 2, 3, 4, 5, 6 and 8 are spontaneous. Their free energy changes were lost as heat. Only 7 has a positive free energy change. The criterion for such a composite but unitary reaction to proceed is simply that there is overall a decrease in free energy in going from reactants to products. Since the process occurred, it is safe to assume that the summation of all 8 free energy changes was negative. There was no need to donate energy to the partial reaction which had a positive free energy change. Heat was absorbed by this reaction, but over all 8 partial reactions, heat is evolved. Obviously for this to be a valid assumption, each partial reaction must contribute to the overall change in free energy.

The significance of analyzing it into partial reactions is that it demonstrates that the free energy changes that allow the overall reaction to happen are delivered in very small quanta, which means that the system is never far from equilibrium and the efficiency of utilisation of free energy is extremely high. If free energy had been released in a single large burst, efficiency would be low and the overall reaction might not proceed. This is one of the wonders of active processes in biochemistry (see the Na,K-ATPase).

### Cellulose acetate as a reverse osmosis membrane

Reverse osmosis is an excellent method of production of water free of ions and most other solutes. Cellulose acetate membranes are still widely and successfully used. A plausible explanation for the mechanism of their action must take into account the properties of the water inside the pores. This water appears to be strongly enriched in LDW. Although it relatively excluded some ions, that exclusion was never absolute. Even MgCl_2_, the most strongly excluded salt, did reach a measurable concentration inside, and KCl had the same concentration inside and out.

There appears to be no acceptable mechanism of action of these very simple membranes. Under reverse osmosis, pressure is applied to the solution side of the membrane. The response is movement of water free of solutes to the other side. The pressure acts upon water on the solution side of the membrane, on areas of membrane between pores and on water inside pores.

According to the estimates of Robinson and coworkers [4]–[6] most water at ambient pressure and temperature is HDW. Therefore, while applied pressure will convert some LDW into HDW in the solution, this will be a slight effect as it will operate only on the relatively sparse microdomains of LDW. Water in the pores, however, is, apparently pure, or, at least; greatly enriched LDW. The effect of applied pressure is therefore to convert all LDW in the pore into HDW.

Here, then, isolated inside the pores, is a near-pure sample of HDW. Since it is not in equilibrium with LDW, as it is in bulk water, there is no thermodynamic constraint preventing it from boiling at its intrinsic boiling point, which Table 1 suggests is well below ambient temperatures. Therefore it instantly vaporises, dropping all its solutes at the mouth of the pore. It is rapidly pushed, in the vapour state, through the membrane pore, emerging at the other end as pure vapour which, out of range of the applied pressure, immediately condenses into the mixture of LDW and HDW determined by the ambient pressure and temperature.

This mechanism gains support from work on carbon nanopores, through which water moves with incredible speed [27]. These pores are less than 2 nm in diameter and bounded by hydrophobic surfaces. They, like cellulose acetate pores, will contain highly enriched LDW. Again the action of pressure is to convert LDW in the pores into HDW which immediately vaporises and moves extremely rapidly through the pores, condensing on the other side. A membrane containing such pores should make an excellent reverse osmosis membrane.

### Polyamide beads [28]–[30]

The density of water in pores of polyamide beads was measured in density bottles, using hexane as a standard. As the size of the pores increased from P-2 to P-6 density of internal water increased [28]. This was, perhaps the greatest legacy of the beads. Using independent data from the manufacturers, it was possible to estimate the thickness of the zones of HDW and LDW. It was estimated (from the manufacturer's exclusion limits) that the pore diameter of P-2 was approximately 2 nm and of P-6, 2–3 nm. At this surface, at least, the zones appeared not to exceed 1–3 nm, because pores with higher exclusion showed little evidence for the existence of LDW. Most experiments used P-4 beads. As a highly concentrated slurry in electrolyte solutions, beads clearly demonstrated the oscillations that had been inferred in cellulose acetate membranes. The mechanism of the oscillations, however, was different. The internal volume of the beads was estimated by including in the external solution polyethylene glycol (MW 3000) labeled with ^14^C. Changes in internal volume of the beads were calculated from the changes in concentration of this molecule as water moved into and out of the pores of the beads with time [29], [30].

When the external solution contained only 10 mM KNO_3_ beads swelled slightly but measurably for 17 days. This suggested that the matrix of the beads was more flexible than that of the cellulose acetate films, so that water could continue to abolish an osmotic pressure gradient without reaching a barrier of inflexibility. When the external solution contained only water, NaCl or MgCl_2_ there was no significant change in internal volume over many days. When, however, the external solution contained both chaotrope and kosmotropes the internal volume oscillated over days. The simplest example was potassium phosphate (pH 7) which consists of the powerful chaotrope KH_2_PO_4_ and the potent kosmotrope K_2_HPO4. Beads swelled and shrank without stopping. This time the mechanism must have been that, initially KH_2_PO_4_ diffused into the LDW in the pores, water followed and the internal volume increased. This continued until the loss of water and KH_2_PO_4_ from the external solution increased the concentration of K_2_HPO_4_ until the osmotic pressure gradient was abolished. Water then stopped moving, but KH_2_PO_4_ continued to accumulate, LDW converted to HDW, and KH_2_PO_4_ and water diffused out. When the pores contained no solutes, LDW reformed and the next cycle began. These movements were controlled by the rate at which the polyamide matrix could adjust itself to influx and efflux of water. The frequency was of the order of two days. [29], [31] Other solutions which supported oscillations were phosphate-buffered saline, culture medium, Ringers solution: all contained high NaCl concentrations and lower concentrations of K^+^.

**Figure 3:**
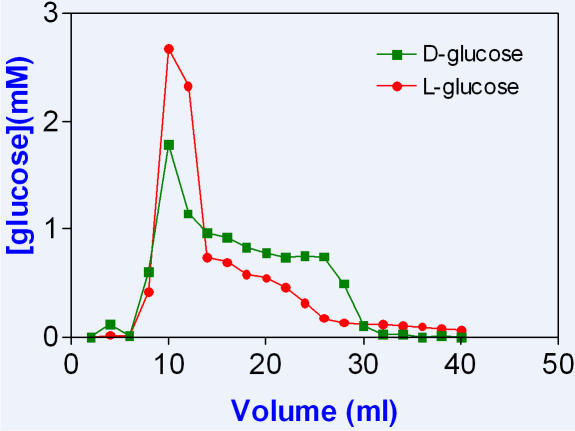
A partial separation of 20 mM D-and 20 mM L-glucose on a P-4 column, prewashed with 100 mM butanol. The eluting solution contained 100 mM butanol.

Polyamide beads both in a column and as a slurry were also used to separate optical isomers of glucose and amino acids. These were very difficult experiments to understand and control. Retention of D-glucose, for example, on a P-4 column, required an external solute excluded from LDW to prevent accumulation of D-glucose from demolishing LDW. The optimal concentration of that solute had to be found by trial and error and was not always the same for different batches of beads. Butanol was found to be a suitable compensating solute. A column was first washed several times with a butanol solution; equal concentrations of D- and L- glucose in the same concentration of butanol, were put on the column and eluted with water. Duplicate experiments with 20 mM L-glucose and 20 mM D-glucose were run: in one D_glucose was labeled with ^14^C, and in the other L-glucose was labeled. Relative retention of D-glucose is shown in Figure 3, in which the concentration of each glucose was 20 mM and the concentration of butanol 100 mM.

**Figure 4:**
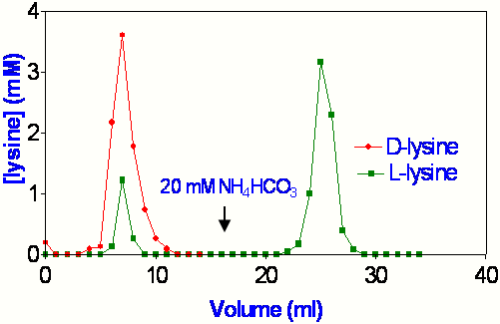
Elution of 20 mM D- and 20 mM L-lysine on a P-4 column, prewashed with 50 mM butanol. The first eluting solution contained 50 mM butanol.

Figure 4 shows a much better separation of 20 mM D- and 20 mM L-lysine in 50 mM butanol. Here, the retained L-lycine was eluted with the powerful chaotrope NH_4_HCO_3_. Other chaotropes used for elution were KNO_3_, KCl and KH_2_PO_4_. L-lysine was labeled with ^14^C and total lysine estimated by adsorption at 214 nm. These two experiments were highly reproducible. In this preparation, at least, the bioactive enantiomorph partitioned into LDW, while the inactive enantiomorph did not.

### Dextran sulphate, glass beads, silica gel and anion and cation exchangers

These polyelectrolyte systems are included under a single heading because their interactions with water and ions are most relevant to biology. Cells contain low concentrations of metabolites and otherwise consist only of polyelectrolytes (soluble and insoluble) and their counterions. The microenvironment determining the properties of water and the behaviour of solutes is the fixed charge and its counterion. Interactions are complex.

**Figure 5:**
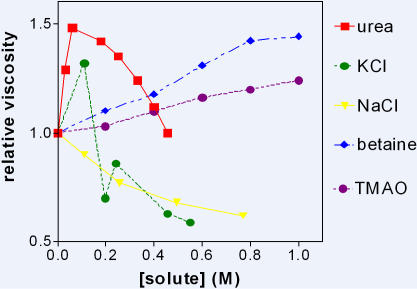
Effects of solutes on the viscosity of dextran sulphate solutions at concentrations of 1 g dextran sulphate in 3 ml water. At higher concentrations of KCl dextran sulphate precipitated.

Figure 5 shows some effects of small solutes on the viscosity of a dextran sulphate solution (3 g water to 1 g dry dextran sulphate). This composition was chosen to resemble that of a cell. The viscosity of the solution was 76 cp. Since the osmotic pressure caused by the counter ion, is not easily abolished by movement of water, the pockets of HDW (in the double layer) and LDW (outside the double layer) are more stable than pockets at uncharged surfaces. Moreover there are more forces to be considered.

The non-electrolytes are simpler to explain than the ions. Nevertheless they show unexpected effects. Urea, for example, steadily increased viscosity up to about 0.5 M when it sharply changed and began to decrease viscosity. It is known that urea partitions into LDW. Presumably, as it partitions into the zone of LDW outside the double layer, it generates an osmotic pressure gradient which is steadily abolished by movement of water out of the double layer into the zone of LDW. This movement is allowed because it reinforces the electrostatic attraction between fixed charge and counter ion. It both increases LDW and decreases HDW, thus increasing viscosity. There is no rigid matrix here to prevent continuation of this process, but it is limited by the need to hydrate the surface and the counter ion. When water stops moving, further accumulation of urea steadily converts some LDW into HDW, thus abolishing the osmotic pressure gradient, and decreasing viscosity by decreasing LDW and increasing HDW.

Trimethylamine oxide (TMAO) and betaine are two highly soluble kosmotropes, according to the present definition. They only moderately partition into HDW. In dextran sulphate solutions, they apparently partition into the HDW in the double layer. The resulting osmotic pressure gradient cannot be abolished by entry of water because of the powerful electrostatic attraction between the fixed charge and counterion. Therefore HDW in the double layer converts steadily to LDW and viscosity increases.

A third non-electrolyte which is not shown in Figure 5, but was extensively investigated, was butanol, a potent kosmotrope. Here an additional effect must be invoked. Butanol was found to behave very much like NaCl in Figure 5: i.e. at all concentrations, and increasing with concentration, it decreased the viscosity of the solution. After the discussion about its fellow weak kosmotropes betaine and TMAO, this is a particular surprise. Presumably butanol was accumulated into HDW in the double layer, and strongly. This must have the effect of decreasing the local dielectric constant in the double layer, and allowing the counterion to move closer to the fixed charge: i.e. thereby thinning the double layer. This concentrated all solutes, increased the osmotic pressure gradient, increased the positive pressure acting on water in the double layer. The overall effect was a stronger enrichment of HDW in the double layer and a decrease in viscosity. Presumably, in this case, the thinning of the double layer due to decreased dielectric constant was greater than its thickening due to influx of water.

While added NaCl had a similar effect to butanol, it was for entirely different reasons. It, too, partitioned into HDW in the double layer, increasing the dielectric constant and increasing the thickness of the double layer. This decreased the concentration of excess solutes, decreased the osmotic pressure gradient and allowed water to enter and abolish the osmotic pressure gradient. This increased the amount of HDW and decreased viscosity.

Discussion of KCl is left until the results of precision glass beads have been discussed.

### Precision glass beads [32]

**Figure 6:**
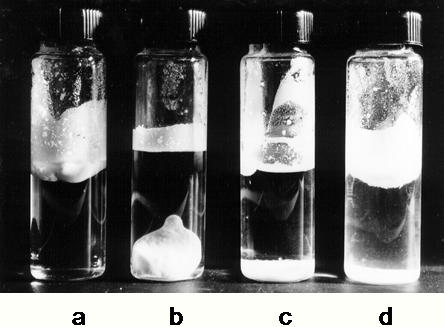
Glass beads (44–60 µm in diameter), made hydrophobic with dimethyldichloro silane. a, 2 g beads in 10 ml water; b, 4 g beads in 10 ml water with countercation K^+^. Cs^+^, Ca ^2+^ or Mg^2+^. c, 2 g beads in 10 ml water with countercation Li+, Na+ or H+; d, the same as c photographed immediately after setting up; the haziness in the water shows the dropping of beads through the water.

The principal objective of these experiments was to explore the effects of counter ion alone on the pockets of LDW and HDW associated with charged groups. The outstanding properties of the hydrophobic beads with K^+^ or Cs^+^ as counter ion were that the beads aggregated strongly, in spite of their negative charges, and floated on water, in spite of their density of 2.6 g/ml. This is illustrated in Figure 6a and b. In 6a, 2 g beads formed a single aggregate floating on the surface of water. In 6b, 4 g beads sank to the bottom of the water, but still as a tightly aggregated mass. Both these properties of the beads must be attributed to formation of LDW at the surfaces. Too much LDW induces aggregation of the beads, decreasing the surface area of contact between beads and water. LDW at the surface also prevented escape of air molecules trapped between the dry beads so that the beads floated. Both aggregation and floating occurred only with water and D_2_O, not with hexane or DMSO. Both aggregation and floating were also absent in uncoated hydrophilic beads.

Fig 6c illustrates the behaviour of hydrophobic beads with Na^+^, Li^+^ or H^+^ as counter ion. The beads formed the single floating aggregate of Figure 6a immediately water was added, but they disaggregated and sank with time. Figure 6d shows beads falling from the floating aggregate immediately after its formation. Clearly these beads were less potent inducers of LDW. The difference must be entirely due to the counter ion.

The greatest surprise was that hydrophobic beads with Ca^2+^ or Mg^2+^ as counter ion formed tighter aggregates and floated more robustly than even those beads with K^+^ or Cs^+^ as counterion.

All these results were consistent with the proposal that enrichment of HDW in the double layer and LDW outside it increased as the counter ion decreased the thickness of the double layer: i.e. when the counterion was either divalent or lightly hydrated.

Erratic changes in viscosity as KCl was added to a dextran sulphate solution are also now understandable. As a counter ion it induced LDW, as a chaotrope it partitioned into LDW and was followed by water at very low concentrations inducing more LDW and decreasing HDW. This is a bad experiment because, in fact two things are happening: KCl is behaving as described, but at the same time Na^+^ is released from its position as counterion and contributing NaCl to the mix.. At just 0.1 M KCl or CsCl, dextran sulphate precipitated out, presumably because its solution now required too great a displacement of the water equilibrium to LDW.

Effects of added nonelectrolytes on glass beads in their Na^+^ form also had the expected effects. Urea at concentrations of 1,2,3 M converted the crumbling aggregate of beads (figure 6c,d) into a tight, floating aggregate. At 6M, however, beads disaggregated. Trehalose, a chaotrope expected to behave like urea, stabilized the Na^+ ^form at 0.2M. TMAO and betaine stabilized the Na^+^ form at 0.5 and 1 M.

### Dowex exchange resins

**Figure 7:**
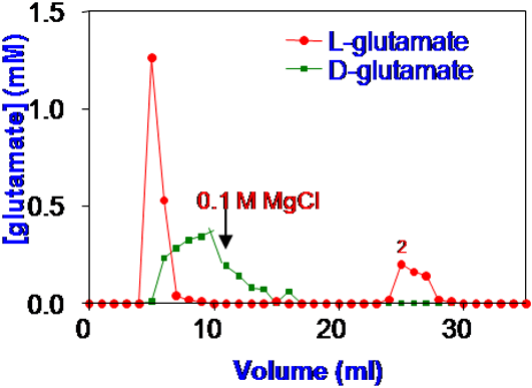
Elution of 20 mM of each of KD-glutamate and KL-glutamate on a Dowex cation exchange resin in its Na^+^ form.

Columns of both anion and cation exchangers, separated enantiomers with and without supporting solutes. Figure 7 shows an example in which D- and L-Kglutamate were separated on a Dowex cation-exchange resin. Since the microenvironments surrounding charged groups stabilized both LDW and HDW, both enantiomers were, to some extent retained on the column. The pattern of elution changed drastically when the counter ion on the resin was changed.

### Silica gel

Silica gels have been used extensively to characterize the properties of surface water, particularly by Drost-Hansen and coworkers who showed that, frequently, there were thermal anomalies in properties measured over a temperature range [33]. The resulting curves were often reminiscent of the abrupt changes observed in many of the experiments with cellulose acetate, polyamide beads and dextran sulphate., suggesting that they, too, reflected changes from HDW to LDW or from LDW to HDW as functions of temperature rather than concentration or time..

### Synthesis of a peptide from L-lysine

Experiments with ion-exchange columns were ambiguous because both L- and D- optical isomers were retained to some extent (although they were then eluted separately with different solutions); the decisive experiment was treatment of a small-pored silica gel with solutions containing D-, L-. and DL-lysine together with the Biuret solution which stains peptide bonds blue. Like the ion-exchangers, a silica gel contains pockets of both LDW and HDW, so that retention of either enantiomer would not establish whether it preferred LDW or HDW. From the experiment in which ATP was synthesized, however, we know that only in LDW would a peptide bond form between two lysines. In this experiment, the sample with only D-lysine gave a scarcely perceptible tinge of blue. The sample with L-lysine stained very strongly as did the sample with DL-lysine. The ability of DL-lysine to form peptide bonds, in spite of the fact that the concentration of L-lysine was only half that in the pure l-lysine solution was due to compensation of the osmotic pressure gradient caused by selective partitioning of L-lysine: D-lysine accumulated outside the LDW generating an osmotic pressure gradient of opposite sign, and protecting the LDW from collapse.

In a similar experiment with D-, L- and DL-glucose in a slurry of polyamide beads, polymers were detected by HPLC in slurries containing D- or DL- but not L-glucose. These experiments cleared up the ambiguity of the column experiments. Polysine and polyglucose could only have been synthesised in LDW.

### The reactivity of HDW

All these polyelectrolyte systems underwent irreversible changes in aqueous solution. Glass beads and silica gel released silicates, a cation exchange resin released HSO_4_^−^, an anion-exchange resin released NH_4_^+^.

Water is an extremely weak acid and base. The concentrations of H^+^ and OH^−^ are each 10^−7^M at 25^o^C. This, like the boiling point of water, is unusual: oxyacids of neighbouring elements in the Periodic Table are often very strong; eg. H2SO4, HNO3, HCl, H3PO4. The first dissociation of strong acids, produces the powerful kosmotrope H^+^, together with a univalent anion (Cl^−^, Br^−^, I^−^ , HSO4^−^, H2PO4^−^, or HCO3^−^) which is a more or less powerful chaotrope. Ionisation is thus possible in both LDW and in HDW, and the osmotic imbalance is so slight that little conversion of either LDW into HDW or of HDW into LDW is required.

The H^+^ ion is the most powerful kosmotrope of all univalent cations; the OH^−^ ion is one of very few kosmotropes among univalent anions. Water ionises, therefore, in HDW which readily dissolves its ionic products but not in LDW. In the mixture of microdomains of bulk liquid water, water is largely unionized, thus avoiding the thermodynamic cost of producing two powerful kosmotropes. In, however, an isolated pocket of water enriched in HDW (such as the double layer of a polyelectrolyte), there is little thermodynamic constraint against ionization, which, producing highly reactive H^+^ and highly reactive OH^−^ at the same time, behaves like a strong acid and a strong base. This is probably how proteases can hydrolyse peptide bonds at ambient pH and temperature. It also accounts for the irreversible changes of the polyelectrolytes in water: it cleaves the bond attaching the charged group to the matrix.

Production of silicates from glass and production of HSO_4_^−^ or NH_4_^+^ from ion-exchangers are accelerated by the unlikely pair of nonelectrolytes urea and butanol. Figure 5 showed that low concentrations of urea partitioned into HDW and was accompanied by water from the double layer. It, therefore, decreased the thickness of the double layer, increasing the concentration of the counter ion, and the pressure resulting from the osmotic pressure gradient. Thus the enrichment of HDW in the double layer increased, as did the rate of cleavage of the sulphate bond. Butanol also thinned the double layer, increasing its enrichment in HDW. But it did that by partitioning into HDW and decreasing the dielectric constant. Mg^2+^ as counterion also thinned the double layer because its two charges held it close to the fixed charge. It greatly accelerated release of silica from glass beads. Other solutes, however, protected against cleavage of the bond between the charged group and the matrix. These included betaine and TMAO, both of which seemed to convert HDW in the double layer of dextran sulphate into LDW, thus abolishing altogether the cleavage of the bond.

## Discussion

All the rather simple systems described have revealed complexities which escaped notice when a narrow range of experimental conditions was used. For example, measurement of the OH stretch band of water in cellulose acetate membranes soaked in a solution of 10 mM KCl was that of liquid water, and there was no significant difference between the concentrations of KCl in the pores and in the external solution. From these measurements alone, the most probable conclusion is that cellulose acetate had no effect at all. Again, 6M urea which denatures proteins, decreased the viscosity of dextran sulphate and disaggregated glass beads in their Na^+^, Li^+^ or H^+^ form: the conclusion from that concentration alone, is that urea is a destructive molecule. Cellulose acetate membranes soaked in solutions containing 100 mM NaCl as well as ADP and KH_2_PO_4_ did not release ATP into the external solution. This, of course, would surprise no-one, and the conclusion is that cellulose acetate membranes did not permit synthesis of ATP.

The history of investigation of water at surfaces and in biochemistry has led to many such generalisations, which are valid for a narrow range of conditions, but totally misleading outside that narrow range.

### Nomenclature

Solutes are traditionally classed as hydrophilic or hydrophobic. The term hydrophilic embraces both Na^+^-like and K^+^-like solutes which, in the mixture model behave in opposite fashion. Hydrophobic implies a hatred of water and is consistent with the extremely low solubility of molecules with many C, CH, CH_2_ groups. In the mixture of HDW and LDW however, that low solubility is attributed to an inordinate love for HDW. Hydrophobic molecules partition with such avidity into HDW that the necessary displacement of the HDW/LDW equilibrium would incur an impossible thermodynamic debt.

The alternative classification, dividing solutes into chaotropes and kosmotropes, which seemed promising initially and has been used above, is at least equally misleading. The definition was given for solutes in bulk water and is accurate for that special case. Experiments, however, have clearly shown that a K^+^-like counterion at a charged surface can increase viscosity, while a Na^+^-like counterion can decrease viscosity. It appears that solutes must be defined, not in terms of their consequences, which are variable, but in terms of their specific invariable partitioning into either HDW or LDW. It also follows that classification of a new solute cannot be made from the result of a single experiment. Whether it partitions into LDW or HDW can only be decided after measurement in more than one local environment.

These are not trivial issues, because false terminology can led to false expectations and false interpretations. Nevertheless, the terminology of chaotropes and kosmotropes will continue to be used here, until a better one is invented. It is essential to remember, however, that it is derived from the single special case of solution in bulk water and that serious deviations can be expected when the concept of polymorphic water is applied to cells [34]–[36].

Reasons for discarding both the terms hydrophilic and hydrophobic and the alternatives chaotropic and kosmotropic are spelt out in these experimental results:

- Butanol is a typical hydrophobic molecule, a typical kosmotrope. Urea is a typical hydrophilic molecule, a typical chaotrope. Those terms all derive from expectations of their behaviour; expectations which are clearly wrong, because butanol and urea both accelerate cleavage of charged groups from polymers.
- Butanol is a strong kosmotrope, betaine and TMAO are less potent kosmotropes, but butanol decreased the viscosity of dextran sulphate solutions while TMAO and betaine increased its viscosity.
- Ca^2+^ and Mg^2+^ are hydrophilic and potent kosmotropes; K^+^ is hydrophilic and a potent chaotrope; Na^+^, Li^+^ and H^+^ are hydrophilic kosmotropes. Ca^2+^, Mg^2+^ and K^+^ induce HDW at charged sites, Na^+^, Li^+^ and H^+^ induce HDW.

## Materials and Methods

### Dense films of cellulose acetate

The films of cellulose acetate that we used carried two acetate groups for each three C-OH groups. The films, themselves, were 4–20 µm thick and their pores about 2 nm in diameter. The surfaces were predominantly uncharged so that ionic selectivity could not be attributed to ion-pair formation. The most important property of these films was that the internal water compartment was easily probed selectively (Wiggins and van Ryn, [24]. Strips of film were soaked in solutions of various chlorides or nitrates, removed, excess bulk water blotted off and the residual film weighed, dried at 110°C and reweighed to give water content. Solutes were then extracted with 0.1 M HNO_3_. We found that up to six days were needed for ions to reach a steady state between internal and external water. This, presumably, was because the cross-linked network of cellulose acetate was extremely slow to expand or contract and allow water and solutes to move in or out. Internal water was highly selective to ions. Pieces of film were also blotted and the OH-stretch band of the infrared spectrum of water measured. In other unpublished experiments pieces of film were soaked in solutions containing 1 mM ADP, 5 mM KH_2_PO_4_, together with 100 mM NaCl, 100 mM MgCl_2_, 100 mM KCl or no added electrolyte. ATP and ADP were measured daily for several days, using HPLC. ATP, was also estimated using the luciferin/luciferase reaction.

### Microporous polyamide beads

Polyamide beads of the BioRad P-series were also a good experimental preparation [26], [27]. They were used either in a column or as a slurry. Again, it was possible to probe a single compartment and obtain surface water properties in the absence of significant surface charges. The density of internal water in variously-sized pores was measured in density bottles and found to decrease as the pore diameter decreased from 6 nm to 1.8 nm. Density of internal water also decreased when beads were equilibrated with increasing concentrations of polyethylene glycol 20M which was too big to enter the pores.

### Dextran sulphate solutions

Dextran sulphate (MW 500,000) is a typical highly charged polyelectrolyte with two sulphate charges for each three glucose monomers. It formed rather viscous solutions, except in the presence of KCl or CsCl when it separated into a gel, containing all the dextran sulphate and a polymer-free electrolyte solution. We measured the effects of added solutes on the viscosity of solutions.

### Precision glass beads

Precision glass beads were obtained from Potters Industries Pty. Ltd., Victoria Australia. Chemical analysis of the beads was : SiO_2_, 72.5%; Na_2_O, 13.7%;CaO, 9.8%; MgO, 3.3%; Al_2_O_3_, 0.4%; FeO/Fe_2_O_3_, 0.2%; K_2_O, 0.1%. The grades used were: B (600–425 µm diameter), AB (300–180 µm diameter) and AH (90–44 µm diameter). They were washed and used as received or treated with Coatasil, 2% dimethyldichlorosilane in 1,1,1-trichloroethane, (AJAX Chemicals Pty Ltd) washed with three volumes of ethanol and six volumes of water, and dried before use. Uncoated beads were also treated with M CaCl_2_, CsCl, KCl, LiCl and HCl, to replace the surface Na^+^ ions with other cations. When these beads were well-washed with water, excess electrolyte was lost and the counter ions were the only associated solutes. Beads in their H^+^ form had reduced charge because some negative charges were protonated and others dissociated. After washing, these beads were also treated with Coatasil.

Dry glass beads were weighed into 40 ml Pierce vials to give a smooth layer on the bottom. Liquid was poured on the beads, the vial gently tipped horizontally and the beads allowed to spread out and float, if they so tended, on the horizontal surface of the liquid. The vial was then returned to a vertical position and beads which had floated on the large horizontal surface remained floating.

### Applications to biological systems

These findings must be applied to biological systems with great care. It becomes easier, however, when it is remembered that cells consist principally of polyelectrolytes. Whether the counter cation to these polyelectrolytes is K^+^ or Na^+^ makes a crucial difference, as illustrated in the glass beads, which disintegrated and sank with Na^+^, but aggregated and floated with K^+^. The normal resting state of a cell has K^+^ as its principal cation. Metabolic rate can be expected to be low as diffusion is slowed down and enzymes are unable to make the conformational changes that are characteristic of their activity. This resting state is converted to a fluid, active state by influx of Na^+^. Oscillation between these two states conserves energy, relative to a single active state.

Before considering specific biochemical reactions it is probably useful to summarise the crucial findings from the simple isolated systems.

1. All solutes appear to partition selectively into either HDW or LDW, generating osmotic pressure gradients.
2. Solutes partitioning into LDW include: K^+^, Rb^+^, Cs^+^, NH4 ^+^, (NR3)4^+^, Cl^−^, HCO3^−^ , H2PO4^−^, HSO4^−^, D-glucose, urea.
3. Solutes partitioning into HDW include: H^+^, Li^+^, H^+^, Ca^2+^, Mg^2+^, betaine, TMAO, L-glucose and compounds rich in C, CH, CH_2_ groups.
4. Neutral salts partition into the type of water preferred by their most potent ion.
5. If water can move to abolish an osmotic pressure gradient, it does so.
6. When swelling of the pocket of water is no longer possible, the osmotic pressure gradient is abolished by conversion of the water in the pocket from LDW to HDW or from HDW to LDW, with efflux of the accumulated solute.
7. Swelling can be prevented by rigidity of the polymeric matrix, by development of an osmotic pressure gradient of opposite sign as water leaves the external solution, or by the electric field holding a counter ion close to a fixed charge. .

### Some biochemical reactions

#### The Na,K-ATPase

The overall action of the sodium pump is:
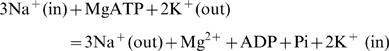
The sequential partial reactions are which have been worked out [18] are:

1. Na^+^ ions enter the active site
2. MgATP enters the cavity
3. ATP phosphorylates an aspartyl residue;
4. Three Na^+^ ions move out into the external solution
5. Two K^+^ ions move into the active site from the external solution.

These reactions must take place in order; i.e. Na^+^ must already be in the site before phosphorylation.

In the light of experiments with polyelectrolytes these partial reactions can be given in more detail.

1. The active site is in its resting configuration with K^+^ as counterion to all charged groups; water immediately adjacent to the surfaces is enriched in HDW; water in the rest of the cavity is enriched in LDW.
2. three Na^+^ ions enter the HDW at the surfaces, replacing three K^+^ ions as counter ions to fixed charges. The cavity becomes more fluid.
3. MgATP enters the cavity
4. Mg ATP phosphorylates an aspartic acid residue, releasing one Na^+^ and replacing it with Mg^2+^ as counteraction.
5. Surface water is further enriched in HDW and the rest of the cavity further enriched in LDW.
6. Enrichment of LDW starts at the phosphorylation site and proceeds upward toward the membrane, pushing the released Na^+^ ahead.
7. A channel at the apex, opening and closing regularly, allows exchange of three Na^+^ ions (two occupying sites near the channel and the third released from the aspartic residue) with two K^+^ ions from the external solution.
8. HDW surrounding the phosphoryl group cleaves the linking bond.
9. The cavity reverts to its resting state with K^+^ions as counterions.

### Comments

It is well known that the ATPase is stimulated by intracellular Na^+^. In the above scheme only free Na^+^ ions that had entered the cell as second messengers, and were in excess of polyelectrolyte fixed charges, would diffuse into the active site.

The power of Mg^2+^ as counter cation is fully exploited by chelating it with ATP. In this way only one ion per ATPase is present. When MgCl_2_ was added to dextran sulphate, viscosity decreased at all concentrations. This mechanism, however, requires a single Mg^2+^ as counterion to generate both enrichment of LDW to push out the Na^+^, and enrichment of HDW to cleave the phosphoryl bond .

The fact that three Na^+^ ions are moved out in exchange for only two K^+ ^ions is necessary for electroneutrality as well as being a well-documented experimental fact. The third Na^+^ does not leave an uncompensated fixed charge. It is itself surplus to electroneutrality requirements in the active site.

Of these reactions, 1,2,4,5,6,7,8,9,10,11 are spontaneous. As in the synthesis of ATP in cellulose acetate membranes, there are sequential small changes, each involving a small release of free energy, making the whole process very efficient. If ATP hydrolysis had happened in a single burst of −30 kJ, it would not have been capable of performing the work of transport. Since the early 1980s [14]–[16] details of this mechanism have changed, as more information from other experiments has surfaced. The most remarkable feature, now, is the power of Mg^2+^ as counter ion to the phosphorylated enzyme. With its double positive charge it is held close to the fixed charge so that the double layer is thin, the concentration of the Mg^2+^ with its co-ion high, and the osmotic pressure gradient extremely high. The result is great enhancement of HDW in the double layer, and development of greatly enhanced LDW in the rest of the cavity. Hydrolysis of the phosphoryl group is a spontaneous process but, because it involves breaking a covalent bond, it is delayed long enough to allow other reactions to occur. The fact that three Na^+^ ions are moved out in exchange for only two K^+ ^ions is necessary for electroneutrality as well as being a well-documented experimental fact. The third Na^+^ does not leave an uncompensated fixed charge. It is itself surplus to electroneutrality requirements in the active site.

The channel through which Na^+^ and K^+^ exchange opens and closes continuously (see next section on channels).

### Ion channels

Ion channels have an entrance compartment which opens and closes, and a filter region which is permanently open to a single ion, thus determining the specificity of the channel.

In the discussion on surfaces it was shown that, depending on the local surface moieties, clefts or pores in proteins can be enriched in either LDW or HDW. If entrance compartments of ion channels are enriched in LDW, they are effectively closed to kosmotropic ions such as Na^+^ and Ca^2+^, but open to K^+ ^and univalent anions. Selective accumulation of K^+^ and anion into LDW in a rigid pore, then converts LDW into HDW. In this state the channel is open to kosmotropic ions. If the filter region is specific for Na^+^, then Na^+^ diffuses spontaneously into the cell, followed by an anion through an anion channel to maintain electroneutrality. K^+^ and anion, which opened the channel for Na^+^, diffuse out into the extracellular solution, having destroyed the LDW which attracted them in. If the driving force for transmembrane K^+^ movement is inwardly directed and the filter specific to K^+^, this might also be a K^+^ channel. Since univalent anions have greater affinity for LDW than has K^+^ they lead the inward diffusion that opens the channels. Experiments have shown, as expected, that electroneutrality must be conserved in the entrance compartment. Many channels are voltage-gated: i.e. they open in response to induction of a more positive membrane potential. Since the anion leads diffusion of a chaotropic ion pair into LDW, too negative a potential would prevent channel opening. With a more positive potential the anion would be attracted in both by its highly favorable partitioning into LDW and by the lowered membrane potential. The cation would follow.

### Evidence that chaotropic ions open channels

Physiologically, intracellular concentrations of Na^+^ and K^+^ are controlled by a steady-state balance between the rates of active Na^+^ efflux and K^+^ influx through the Na,K-ATPase and rates of passive Na^+^ influx and K^+^ efflux through selective channels. The Na,K-ATPase is inhibited specifically by ouabain, by depletion of intracellular ATP and by absence of either intracellular Na^+^ or extracellular K^+^. When cells and tissues are treated with ouabain or anoxia, they lose intracellular K^+^ and take up NaCl and water. With time this swelling becomes irreversible and cells die. Presumably, the passive channels are still opening and closing, but there is no active efflux of Na^+^ or influx of K^+^. When, however, the same cells are treated with a K^+^-free medium, cells do not swell and do not take up NaCl. It is known, from experiments with isolated membranes that, under these conditions, active influx of K^+^ and efflux of Na^+^ do not take place. That cells fail to swell and take up NaCl must, therefore, mean that, in the absence of K^+^, passive channels are also inhibited.

### Ca^2+^- activated K^+^ channels

A channel filled with HDW is open to Ca^2+^ but closed to K^+^. Such a channel could be opened by CaCl_2_ which converts HDW to LDW, thus allowing passage of K^+^.

### Preservative solutions

We have used this concept of channel-opening to design solutions in which cells achieve a state of dormancy, and can survive without energy input for days or weeks. Solutions contained no channel-opening ions such as K^+^ and univalent anions other than Cl^−^. Osmolality of 290 mOsM/kg was made up with NaCl, betaine, TMAO, all excluded from LDW and raffinose (excluded from the entrance compartment by size). Solutions were first tested using murine embryos. The end point was whether or not the embryos progressed to hatching in culture after preservation in such solutions. [37]. Embryos survived for 5–6 days in some preservative solution, compared with 1–3 days in a balanced saline solution. The rationale was that, with all channels closed, embryos remained in a resting state and their energy consumption was slight .

### Mineralisation of bone

#### Phosphates

The phosphate species have many important properties in polymorphic water;

1. PO_4_^3−^ partitions very strongly into HDW;
2. HPO_4_^2−^ partitions strongly into HDW
3. H_2_PO_4_^−^ partitions very strongly into LDW

These three species are intimately involved in synthesis and dissolution of bone. Notice that a rather slight change in pH (at about 7) converts a powerful chaotrope (H_2_PO_4_^−^) into a powerful kosmotrope (HPO_4_^2−^).

#### Calcium

Ca^2+^ partitions very strongly into HDW. A general rule is that the more highly charged is an ion the more strongly it partitions into HDW.

In order that bone may form in blood, there must be sequential steps:

1. Ca^2+^ and PO_4_^3−^ must locally concentrate near existing bone
2. The concentrated Ca_3_(PO_4_)_2_ must crystallise on the surface of existing bone
3. There must be a mechanism to prevent continuing proliferation of bone

#### Concentrations of Ca^2+^ and PO_4_^3−^

The surface of bone, with Ca^2+^ ions countercations to phosphates is highly likely to have a configuration in which HDW exists in the double layer at the immediate surface, with a zone of LDW further out. Since the surrounding medium contains K^+^ and anionic chaotropes, the LDW is not constantly present, but forms, collapses and reforms. Thus the HDW is accessible to solutes which partition preferentially into it. These solutes are Na^+^, Ca^2+^, H^+^ and HPO_4_^2−^. PO_4_^3− ^is not included because its concentration is too low for it to be a significant player. As, however, HPO_4_^2−^ accumulates into HDW in the double layer, it is rapidly converted into PO_4_^3− ^by the equation:
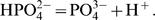
(i.e. a rather potent kosmotrope converts spontaneously to two very potent kosmotropes in the HDW). These concentrating electrolytes increase the dielectric constant in the double layer, allowing the Ca^2+^ ion to move a little away from the phosphate on the surface, thus increasing the thickness of the double layer by movement of water from the adjacent zone of LDW. The species which we want to crystallise, increase locally in amount.

Ca_3_(PO)_4_ is less likely to crystallise in HDW than it was in the mixture of waters. Something is needed, therefore, to stop the flow of water from the adjacent LDW zone so that water in the double layer converts to LDW. When this happens, Ca_3_(PO)_4_ immediately crystallises on the surface of bone. Following crystallisation, remaining kosmotropes diffuse out and water in the double layer resumes its composition of highly enriched HDW. Another cycle begins. The trigger for stopping the flux of water must come from osteoblasts. It seems most probable that the trigger is type 1 collagen which is secreted by osteoblasts. The most frequent amino acid residues in collagen are proline, glycine and alanine [12].

Like butanol in dextran sulphate solutions, therefore, collagen accumulates into the enriched HDW in the double layer; the many hydrophobic patches along the molecule decrease the dielectric constant and thin the double layer. The folded collagen which entered the HDW opens out to a degree, increasing the surface area between water and hydrophobic patches so that thinning of the double layer is extreme. The mechanism for this is that, protein folding is driven by the need to avoid some of the cost of displacing the water equilibrium from HDW to LDW. This cost has a term in-RTlnN_H_/N_L,_ where N_H_ is the mol fraction of HDW and N_L_ the mol fraction of LDW. Therefore the cost to be avoided and the driving force for folding decreases as the mol fraction of HDW increases. Thus the collagen which folded to a triple helix of low entropy in the mixture HDW/LDW can unfold, to a degree, in highly enriched HDW. This increases its tendency to thin the double layer. As the double layer decreases in volume, the concentrations of the accumulated ions, Ca^2+^ and PO_4_^3−^ increase; water cannot abolish the resulting osmotic pressure gradient; HDW switches to LDW and Ca_3_(PO)_4,_ entangled with collagen, crystallizes as bone. HDW reforms in the double layer.

The collagen which precipitates in LDW does not redissolve because it is not correctly folded for solubility in bulk water.

### Regulation of growth of bone

The continuous production of bone by osteoblasts is matched by a continuous dissolution of bone by osteoclasts, so that a steady state can be reached. This is similar to the steady state of intracellular Na^+^ which is maintained by means of approximately equal numbers of pumps which eliminate it and channels that allow it to diffuse spontaneously in. The plasma membranes of osteoblasts carry proton pumps which acidify the adjacent bone. This converts PO_4_^3−^ into the highly soluble H_2_PO_4_^−^, dissolving the bone locally. The steady state of bone is modified when net growth is needed, sustained when growth is no longer needed but, like many steady states, declines with age.

### To end at the beginning: a possible chemical origin of life

Primordial clays carried both positive and negative charges with counterions, Na^+^, K^+^, Al^3+^, Ca^2+^, Mg^2+^. Hexoses and amino acids were among the many small organic molecules released into space from exhausted stars long before our galaxy formed [38]. A chemical start to life, therefore can assume their presence, but must have some way of overcoming the inability of the monomers to join together. The search has been for heat to supply energy for these reactions. That, however, is no longer necessary. All that was needed was a change of solvent. That was probably supplied in the double layers associated with polyelectrolyte clays. The monomers joined together in the solvent LDW and were released to the solvent LDW/HDW by the third solvent, reactive HDW. And, for good measure, only one of the two enantiomorphs formed polymers.

### Conclusion

Maybe polymorphic water has enough explanatory power to contribute significantly to our understanding of biochemistry and its beginnings.
